# Flow-Batch Sample Preparation for Fractionation of the Stress Signaling Phytohormone Salicylic Acid in Fresh Leaves

**DOI:** 10.1155/2020/8865849

**Published:** 2020-07-17

**Authors:** Thiago L. Marques, Milton K. Sasaki, Lidiane C. Nunes, Fábio R. P. Rocha

**Affiliations:** ^1^Center for Nuclear Energy in Agriculture, University of São Paulo, Av. Centenário, 303, 13416-000 Piracicaba, SP, Brazil; ^2^Federal University of Rio Grande do Sul, Avenida Bento Gonçalves, 91540-000 Porto Alegre, RS, Brazil

## Abstract

Salicylic acid (SA) is an important stress signaling phytohormone and plays an essential role in physiological processes in plants. SA fractionation has been carried out batchwise, which is not compatible with the high analytical demand in agronomical studies and increases susceptibility to analytical errors. In this context, a novel flow-batch sample preparation system for SA fractionation on fresh plant leaves was developed. It was based on microwave-assisted extraction with water and conversion of the conjugated species to free SA by alkaline hydrolysis. Free and total SA were quantified by fluorimetry after separation by sequential injection chromatography in a C18 monolithic column. The proposed procedure is directly applicable to plant leaves containing up 16 mg kg^−1^ SA, with a limit of detection of 0.1 mg kg^−1^ of SA, coefficient of variation of 3.0% (*n* = 10), and sampling rate of 4 samples h^−1^. The flow-batch sample preparation system was successfully applied to SA fractionation in sugarcane, corn, and soybean leaves without clogging or increasing in backpressure. The proposed approach is simple, less time-consuming, and more environmentally friendly in comparison to batchwise procedures.

## 1. Introduction

Salicylic acid (SA) is an important phytohormone in plants, which acts as stress signaling, as well as on seed germination, seedling formation, plant respiration, legumes nodulation, fruits production, and defense against pathogens [1–3]. Although free SA is the active form involved in physiological processes, it is stored in cellular vacuoles either as inactive glycosylated or methylated species [3]. Thus, fractionation of SA is relevant to understand plant response under stressing conditions and to evaluate the activation of the mechanism of the plant defense [4].

Determination of SA and its derivatives in plants demands selective and sensitive procedures because the species are usually found in low contents (mg kg^−1^) in a relatively complex matrix [5, 6]. Chromatographic techniques are usually applied for this task, including high performance liquid chromatography (HPLC) with detection by mass spectrometry (MS) [6], tandem mass spectrometry (MS/MS) [7], fluorimetry [8] or spectrophotometry [9], ultra high performance liquid chromatography coupled to MS/MS [4], or gas chromatography (GC) coupled to MS [1, 10]. These alternatives are relatively expensive and sample preparation involves several steps, which increase sample handling and susceptibility to analytical errors [4, 6, 7]. In addition, these procedures typically consume large amounts of solvents and generate large quantity of residues, which is not in accordance to the principles of Green Analytical Chemistry [11].

Sequential injection chromatography (SIC) combines sequential injection analysis (SIA) with monolithic or fused-core particles columns operating at relatively low pressures to promote fast and cost-effective chromatographic separations, minimizing eluent volumes and waste amounts [12, 13]. Despite the lower chromatographic efficiency compared to HPLC, successful separations have been achieved for a diversity of applications, including determination of pharmaceutical compounds in medicines [12] and body fluids [14], contaminants in food [13], and pesticides in natural waters [15]. Barrientos et al. [16] successfully applied SIC with fluorimetric detection for determination of free SA on plant leaves after batchwise microwave-assisted extraction. It was demonstrated that fluorimetry based on the intrinsic fluorescence of salicylate is a cost-effective alternative to the MS detectors. Other studies have demonstrated that the conjugated species can be quantified after conversion to salicylate either by acid [8] or alkaline [17] hydrolysis, which allows fractionation of SA. A systematic evaluation of the batchwise experimental conditions for SA fractionation in plant leaves was recently presented [18].

Analyte extraction from solid samples has been predominantly performed on the batch mode, which is typically time-consuming and increases susceptibility to analytical errors. Alternatively, some flow systems have been successfully applied for this task [19], including bioavailability studies [20]. Applications involving treatment of plant tissues prior to chromatographic determination have also been reported [21–23]. As an example, Wu et al. [23] developed a microwave-assisted extraction flow system for pesticide determination on vegetables by GC-MS. Samples were mixed with sand and packed into a glass column, which was put inside a domestic microwave oven. After extraction with a NaCl solution and online single drop microextraction with toluene, the pesticides were quantified into the organic phase by GC-MS [23]. The need for packing each sample into the glass column, the risks of clogging, and increase of backpressure are drawbacks that can be overcome by flow-batch extraction systems [24]. This approach combines advantages of flow-based (e.g., high sample throughput, low reagent consumption, and continuous system washing) and batchwise (e.g., better sample to reagent interaction and long sample residence times) procedures [25]. The potentiality of flow-batch extraction systems is increased by exploiting flow programming inherent to SIA, which allow independent handling of reagents, flow rates, and timing.

In this work, a flow-batch approach was combined to SIA for SA fractionation on plant tissues. The proposed system allows microwave-assisted extraction from the solid samples followed by either the direct determination of free SA or online alkaline hydrolysis aiming at the determination of total SA. The analytical determinations were carried out by fluorimetric detection of the salicylate ion after separation of the sample matrix by sequential injection chromatography.

## 2. Experimental

### 2.1. Apparatus

Extraction of the analytes from the plant tissues and hydrolysis of the conjugated forms of SA were performed with a SIA system (FIAlab® 3500, FIAlab Instruments Inc.®, Bellevue, USA) equipped with a 10 mL syringe pump, a bidirectional peristaltic pump, and an eight-port low-pressure PPS selection valve (Valco Instrument Co., Houston, USA). Analyte separation was performed with a SIChrom™ system (FIAlab Instruments®, Bellevue, USA) with an eight-port high-pressure stainless-steel selection C5H valve (Valco Instrument Co., Houston, USA) and an S17 PDP syringe pump (Sapphire™ Engineering, MA, USA) with a 4.0 mL reservoir. Flow systems were controlled by a microcomputer equipped with FIAlab® 5.9 software (FIAlab Instruments®). PTFE (0.8 mm i.d.) and PEEK (0.25 mm i.d.) tubing were used in SIA and SIC systems, respectively. Chromatographic separations were performed on a monolithic column (Phenomenex® Onyx™, C18, 50 mm long and 4.6 mm i.d.) with a 5 mm long guard column. Measurements were carried out with a spectrofluorimeter (Varian, Eclipse, Mulgrave, VIC, Australia) equipped with a 40 *μ*L quartz flow cell. The excitation and emission slits were both adjusted to yield a 20 nm resolution and the software provided by the manufacturer was used for data acquisition.

A domestic microwave oven (2450 MHz, 700 W, NN-ST254W, Panasonic, Manaus, Brazil) was used for the flow-batch microwave-assisted extractions of SA and its derivatives, and a water bath with a 1 mL glass chamber was used for hydrolysis of the conjugated forms to free SA. A magnetic stirrer (Mod 753A, Fisatom, Brazil) with temperature control was used to heat the water bath and stir solutions inside the glass chamber. A cryogenic mill (Spex Model 6870, USA) with a self-container liquid nitrogen bath was used for sample grinding.

### 2.2. Reagents and Solutions

All solutions were prepared with deionized water (resistivity > 18 MΩ cm), analytical grade reagents, and HPLC grade solvents (Merck, Germany). Working solutions from 0.05 to 2.00 mg L^−1^ salicylic acid were prepared by dilution of a 1.000 g L^−1^ stock solution in water.

Alkaline and acid solutions used for hydrolysis and extract neutralization were prepared from NaOH and HCl (both from Merck, Germany). A 0.8 mol L^−1^ acetate buffer solution (pH 5.0) was prepared from sodium acetate (Sigma-Aldrich, USA) with pH adjusted to 5.0 with 1.0 mol L^−1^ HCl solution. The mobile phase for the chromatographic separation was a 7% v/v acetonitrile in 0.2 mol L^−1^ acetate buffer solution (pH 5.0) [16].

### 2.3. Samples

Corn, sugarcane, and soybean leaves were collected from farmers at Piracicaba and Santa Cruz do Rio Pardo, SP, Brazil. Fresh leaves were washed with deionized water, dried at room temperature between paper sheets, grinded in a cryogenic mill for 15 min, and stored in polyethylene flasks in a domestic freezer at −20°C. Just before analyses, samples were stabilized to ambient temperature.

### 2.4. Flow Diagram and Procedure

**Figure 1:**
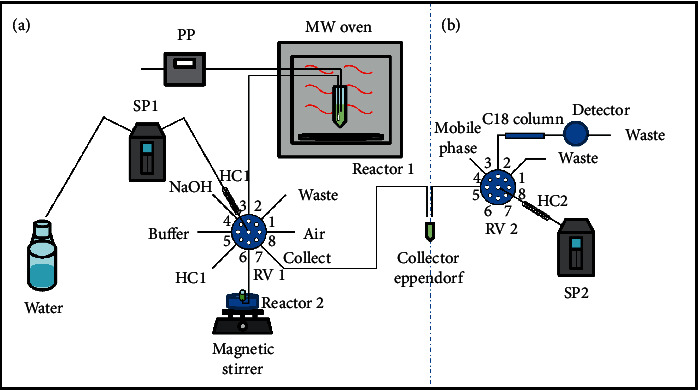
Diagram of the flow-batch system applied to SA fractionation on plant leaves. (a) Microwave-assisted extraction and alkaline hydrolysis. (b) Sequential injection chromatographic system. PP: peristaltic pump; SP1 and SP2: syringe pumps; RV1 and RV2: 8-way rotary valves; HC1 and HC2: holding coils.

The SIA system for online solid sample preparation (Figure 1(a)) was operated as described in Table 1. The grinded sample (500 mg) was introduced into a 50 mL Falcon® tube, which was placed in the center of the microwave oven and used as the flow-batch chamber (extraction reactor). This reactor was connected to the SIA system through two 70 cm long PTFE tubes trespassing the oven wall, for addition of the extractor (deionized water) and aspiration of the extract. A 100 *μ*L micropipette tip with filter was adapted to the aspiration tube for filtration of the extract, as previously proposed [25].

Microwave-assisted flow-batch extraction was carried out with 2 mL of deionized water introduced in the tube by the peristaltic pump of the SIA system (30 s, 4 mL min^−1^). The tube was heated during 6.5 min at 140 W, which corresponds to 20% of the maximum microwave power, achieving a final temperature of (54.5 ± 0.5)°C. An aliquot of the extract was used for channel washing prior to collecting and preparing crude and hydrolyzed extracts. Air was used as carrier to minimize cross contaminations and to avoid sample dispersion. For determination of free SA, 100 *μ*L of the extract was aspirated to the holding coil (HC1) together with 50 *μ*L of 0.8 mol L^−1^ acetate buffer (pH 5.0), 25 *μ*L of 0.5 mol L^−1^ NaOH, and 25 *μ*L of 0.5 mol L^−1^ HCl for adjustment of pH and ionic strength. Then, the sample zone was transferred to an Eppendorf® tube for posterior determination by SIC. Subsequently, HC1 was washed with water to prepare the hydrolyzed extract for determination of total SA. Then, 100 *μ*L of the extract and 25 *μ*L of 0.5 mol L^−1^ NaOH were aspirated to HC1 and transferred to the hydrolyzer reactor placed inside a water bath at (80 ± 2)°C. The solution was stirred for 5 min and the hydrolyzed extract was aspirated to HC1, followed by 25 *μ*L of 0.5 mol L^−1^ HCl and 50 *μ*L of 0.8 mol L^−1^ acetate buffer (pH 5.0). Further, the sample zone was transferred to an Eppendorf® tube for total SA determination by SIC.

Crude and hydrolyzed extracts were analyzed with the SIC system (Figure 1(b)) as described by Barrientos et al. [16]. Initially, 3.9 mL of the mobile phase was aspirated to HC2 at 50 *μ*L s^−1^ followed by 10 *μ*L of the extract. The sample zone was pumped at 10 *μ*L s^−1^ through the chromatographic column and spectrofluorimetric detection was performed with excitation and emission at 298 and 406 nm, respectively. Measurements were based on peak areas and carried out in duplicate (system optimization) and triplicate (SA fractionation).

### 2.5. System Optimization

Multivariate optimization was carried out to maximize free and total SA peak areas, which are indicative of the efficiency of analyte extraction and hydrolysis. A sample of sugarcane leaves with a total SA content of 0.4 mg kg^−1^ previously determined by a reference procedure [18] was used in the optimization step. Maximum and minimum levels of each variable were chosen based on the conditions set for batchwise extraction of SA and hydrolysis of the conjugated forms [18]. The experimental data were processed using the STATISTICA 8.0 software.

A two-level full factorial design (2^3^) was performed to evaluate the effect of the parameters involved in the flow-batch alkaline hydrolysis, that is, hydrolysis time, NaOH concentrations, and temperature (Table S1). Experiments were carried out using 100 *μ*L of the sugarcane leaves extract, prepared from 2.0 g of sample and 8 mL of water according to a batchwise procedure [16]. The peak areas of total SA were taken as indicative of the hydrolysis efficiency. A Doehlert matrix design [26] was used for optimization of the flow-batch microwave-assisted extraction. The extraction time (*X*_1_) was evaluated in five levels (2.0, 3.5, 5.0, 6.5, and 8.0 min) and microwave power (*X*_2_) was evaluated in three levels (20, 40, and 60%), Table S2. The replicates at the central point were exploited to estimate the experimental, pure, and lack of fit errors. The robustness of the flow-batch procedure under the optimized conditions was evaluated by a 2^3^ full factorial design (hydrolysis step) and by the univariate method (extraction step).

## 3. Results and Discussion

### 3.1. General Aspects

**Figure 2:**
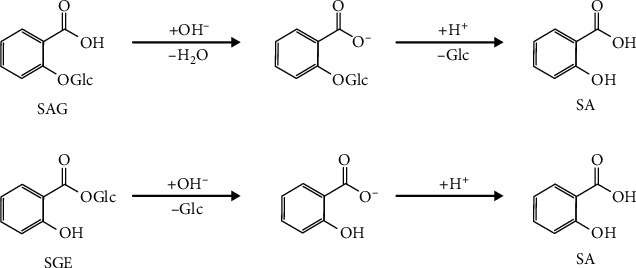
Scheme of chemical reactions involved in the hydrolysis of SA derivatives. SAG: salicylic acid *O-β*-glucoside; SGE: salicyloyl glucose ester; SA: salicylic acid.

The fractionation method exploited in this work is based on the measurement of the intrinsic fluorescence of salicylate ion. Free SA can be directly measured in plant extracts, whereas the total amount is measured after conversion of the conjugated forms to free SA by alkaline hydrolysis [17, 18]. Salicylic acid *O-β*-glucoside (SAG) and salicyloyl glucose ester (SGE) are examples of SA derivatives [3] that are converted into SA after alkaline hydrolysis and solution acidification as shown in Figure 2.

**Figure 3:**
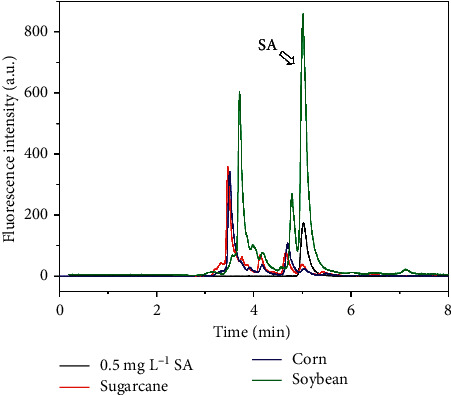
Chromatogram of extracts of sugarcane, corn, and soybean leaves obtained by sequential injection chromatography. The response for a SA standard is shown for comparison. Sample volume = 10 *μ*L, mobile phase = 7% v/v acetonitrile in 0.2 mol L^−1^ acetate buffer solution (pH 5.0), flow rate = 10 *μ*L s^−1^.

The conjugated species are then quantified by subtracting the concentration of free SA from the total SA content. Chromatographic separation is required to avoid interferences on determination of salicylate, caused by either other fluorescent species or quenchers in the sample extracts. SA was efficiently separated from the sample matrix of extracts of sugarcane, corn, and soybean fresh leaves by SIC (resolution >1.6 for adjacent peaks) and quantified in retention time of 5 min (Figure 3).

The literature reports different solvents such as water [16], methanol plus ethyl acetate [1], chloroform [10], methanol [1, 4] or ethanol [9], and temperatures (from −20°C to 80°C) [5, 8, 9, 16] for extraction of SA from plant materials. Although convective heating [8] has been exploited, microwave-assisted heating proved to be a reliable and fast approach for extraction of SA and its derivatives using greener solvents, such as water [16] and ethanol/water solutions [6, 18]. In fact, extraction efficiencies were up to 20 and 40% higher for free and total SA, respectively, by comparing microwave-assisted process to convective heating [18].

Alkaline hydrolysis is slower in ethanolic medium than in water, demanding 60 and 5 min to achieve the steady-state, respectively [18]. This is a critical aspect for online hydrolysis of the conjugated forms because of the compromise between the residence time and sample throughput. Based on these aspects and aiming at a more environmentally friendly procedure, water was preferred as extractor in the flow-batch system.

### 3.2. System Optimization

**Figure 4:**
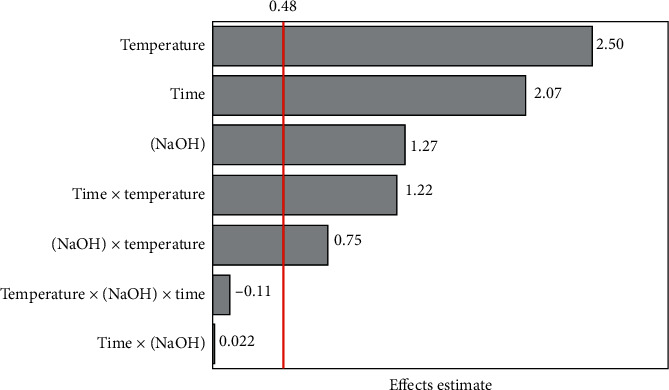
Pareto chart corresponding to the optimization of flow-batch alkaline hydrolysis of the conjugated forms of SA.

A two-level full factorial design was carried out in an exploratory study to evaluate the effects of hydrolysis time (*X*_1_), NaOH concentration (*X*_2_), and temperature (*X*_3_), as well as their interactions, on flow-batch alkaline hydrolysis. Peak areas related to total SA were taken as the analytical response (*y*). From the Pareto chart (Figure 4), it can be concluded that, at 95% confidence level, all variables evaluated as well as the time-temperature and NaOH concentration-temperature interactions affected significantly the hydrolysis efficiency:

(1) $$\begin{array}{c} y=2.12+1.03X_{1}+0.64X_{2}+1.25X_{3}+0.63X_{1}X_{3}+0.38X_{2}X_{3}. \end{array}$$

The effect of temperature was the most significant. Increasing hydrolysis efficiency with temperature was expected by considering the limited sample residence time and agreed with the results observed in batchwise hydrolysis [18]. The relatively slow hydrolysis of the conjugated species was also evidenced by the positive coefficient related to the reaction time (*b*_1_ = +1.03). Hydrolysis is also favored at higher NaOH concentration (*b*_2_ = +0.64). Moreover, effects of interactions between time and temperature (*b*_13_ = +0.63) and NaOH concentration and temperature (*b*_23_ = +0.38) indicate that hydrolysis is favored when all variables are in the highest levels, which correspond to experiment 8 (Table S1). This working condition (0.1 mol L^−1^ NaOH and hydrolysis at 80°C for 5 min) was selected for further experiments. Despite the full factorial design indicated that a higher response would be achieved, the variables were not evaluated at higher levels because increase of NaOH concentration, time, and temperature could damage the chromatographic column, hinder sample throughput, and promote losses of the solvent and volatile derivatives (e.g., methyl salicylate), respectively.

Preliminary experiments indicated that both microwave power and extraction time affected the extraction of the analytes [18]. Doehlert design was then used to optimize the microwave-assisted extraction of SA and its derivatives from sugarcane leaves (Table S2 shows the seven experiments required). Peak areas for free and total SA were both evaluated as the extraction conditions for the different species may differ significantly. Variation in peak areas for free SA was not significant at 95% confidence level, indicating that any region of the established experimental domain would yield similar results. On the other hand, the regression model for total SA was statistically significant, as evaluated by the analysis of variance (ANOVA), Table S3. In fact, by comparing the Mean Square of Regression (MSR) and Mean Square of Residual (MSr), the *F* value (*F*_cal_ = 53.2) was higher than the tabulated value (*F*_tab_ = 9.01) at the 95% confidence level. Moreover, comparing the ratio between Mean Square of lack of fit (MSlof) and Mean Square of pure error (MSpe), *F*_cal_ = 0.50, which is lower than *F*_tab_ = 18.5, indicating that the proposed model does not present lack of fit, at the 95% confidence level. The good adjust of the proposed model is also evidenced by the coefficient of determination (*R* > 0.99).

The regression model for the microwave-assisted extraction is described by (2) that presents six coefficients (*b*_0_ (constant), *b*_1_ (extraction time), *b*_2_ (microwave power), *b*_1_^2^, *b*_2_^2^, and *b*_1_ × *b*_2_) and their confidence intervals (95% confidence level) between parentheses:

(2) $$\begin{array}{c} y_{\text{total SA}}=9.75\left(\pm 0.76\right)+1.32\left(\pm 0.76\right)X_{1}-1.77\left(\pm 0.76\right)X_{2}-1.96\left(\pm 1.20\right)X_{1}^{2}+1.13\left(\pm 1.20\right)X_{2}^{2}-1.95\left(\pm 1.52\right)X_{1}X_{2}. \end{array}$$

**Figure 5:**
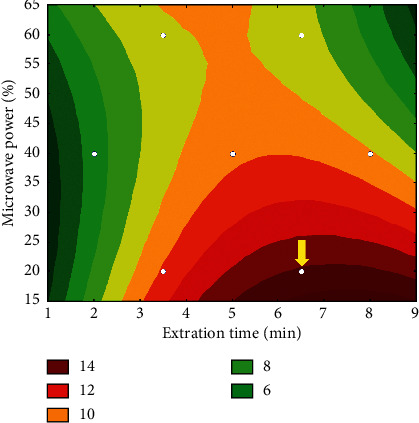
Contour plot corresponding to the microwave-assisted extraction of total SA from plant leaves.

In order to maximize peak areas for total SA, the extraction time and the microwave power should be at the highest and lowest levels, respectively. In addition, interaction between these variables was significant. The contour plot of the proposed model is shown in Figure 5, in which the arrow indicate the optimal condition for determination of total SA in plant leaves, that is, extraction with 20% microwave power for 6.5 min that corresponds to the experiment 5 (Table S2).

### 3.3. Analytical Features and Application

A linear response was achieved from 0.05 to 2.0 mg L^−1^ of SA, described by the equation peak area = (84 ± 3) (SA) − (0.86 ± 0.03), *r* = 0.999, in which (SA) refers to salicylic acid concentration in mg L^−1^. This linear response range corresponds to 0.4 to 16 mg kg^−1^ SA in the plant leaves under the set conditions. The limit of detection (LOD) was estimated as 0.01 mg L^−1^ (equivalent to 0.1 mg kg^−1^ of SA in fresh samples) according to IUPAC recommendations [27]. The coefficient of variation was 3.0% (*n* = 10) and variations of the analytical response between days were lower than 5.0%. The proposed procedure consumed only water for extraction of the analytes and as low as 1 mg NaOH, 2 *μ*L of 37% m/v HCl, and 6.6 mg of sodium acetate per sample for SA fractionation. The waste volume for determination of both fractions is 2.2 mL and sample throughput was estimated as 4 h^−1^. For comparison, the batch procedure for SA fractionation [18] consumes 6 mL of ethanol, 2 mg NaOH, 4 *μ*L of 37% m/v HCl, and 0.5 *μ*L H_3_PO_4_ 85% m/v per sample and generates 9.6 mL of waste per sample. It also demands 60 min to promote the alkaline hydrolysis, in comparison to 5 min in the flow-batch procedure. However, a set of extracts can be simultaneously hydrolyzed in the batch procedure. Consumption of acetonitrile per determination was 10-fold lower in SIC in comparison to HPLC [18].

A robustness test was performed using a 2^3^ factorial design with variation of ±10% in relation to the optimum parameters defined on the hydrolysis step, that is, 0.1 mol L^−1^ NaOH and 5 min at 80°C. The ANOVA from the results in Table 2 indicated that the procedure is robust in relation to the hydrolysis step.

The robustness test related to the extraction step was carried out in a univariate manner because it was not possible to range microwave power on 10% in a commercial oven in relation to optimum condition (i.e., 18 and 22%). Results obtained for microwave power of 10 and 20% and extraction time from 6.0 to 8.0 min did not differ significantly at 95% confidence level. Thus, the flow-batch system, under the optimized conditions, is also robust in relation to the extraction step.

For accuracy assessment, sugarcane, corn, and soybean leaves previously analyzed by batch procedures for free [16] and total SA [18] determination were assayed by the flow-batch sample preparation procedure (Table 3). The mean values agreed and the variances were not significantly different, according to statistical tests (*F*-test and paired *t*-test) both at the 95% confidence level. Acceptable precision was achieved for the sample extracts, with CV from 2.6 to 17% (Table 3). The high contents of free SA on soybean leaves were expected because these plants were on development, while sugarcane and corn were mature plants.

## 4. Conclusions

The proposed flow-batch sample preparation system is a reliable approach for online fractionation of SA on plant leaves. System was able to handle solid samples in a sequential injection analysis system without clogging and using ordinary laboratorial devices (flasks and a pipette tip with filter). All sample preparation steps were performed in a closed system, which avoids analytical errors due to sample contamination and analyte losses. The flow-batch approach was successfully exploited to improve interaction of the solid sample with the extractor as well as providing the required residence times needed to analyte extraction and hydrolysis. Extractions were carried out with deionized water and minimal reagent amounts were consumed in the hydrolysis of the conjugated species, which are in agreement with Green Analytical Chemistry principles. Fractionation of each sample takes about 15 min, which is in compliance with the high analytical demand for SA fractionation in agronomic studies. The developed procedure is a simple, environmentally friendly, and suitable alternative for routine analysis, which is potentially useful for other applications involving microwave-assisted extraction and analyte fractionation.

## Figures and Tables

**Table 1:** Analytical routine for sample preparation in the flow-batch system.

Step	Valve of syringe pump	Selection valve port	Time (s)	Volume (*μ*L)	Flow rate (*μ*L s^−1^)
*Microwave-assisted extraction*					
Extractor insertion into reactor^*∗*^	—	—	30	2000	67
Microwave irradiation	—	—	390	—	—

*Washing of the extraction channel*					
Carrier aspiration	In	—	5	1000	200
Extract aspiration	Out	2	12	600	50
Discard to waste	Out	1	8	1600	200

*Crude extract preparation*					
Carrier aspiration	In	—	5	1000	200
Air aspiration	Out	8	10	100	10
Extract aspiration	Out	2	10	100	10
Buffer aspiration	Out	4	5	50	10
HCl aspiration	Out	5	2.5	25	10
NaOH aspiration	Out	3	2.5	25	10
Extract collection	Out	7	37	370	10
Delay	—	—	20	—	—
Channel washing	Out	7	4.65	930	200
Air aspiration	Out	8	10	200	20
Channel empty	Out	7	10	200	20

*Hydrolyzed extract preparation*					
Carrier aspiration	In	—	5	1000	200
Air aspiration	Out	8	10	100	10
NaOH aspiration	Out	3	2.5	25	10
Extract aspiration	Out	2	10	100	10
Transport towards hydrolysis chamber	Out	6	28	280	10
Hydrolysis time	—	—	300	—	—
Hydrolyzed extract aspiration	Out	6	28	280	10
HCl aspiration	Out	5	2.5	25	10
Buffer aspiration	Out	4	5	50	10
Hydrolyzed extract collection	Out	7	37	370	10
Delay	—	—	20	—	—
Channel washing	Out	7	4.65	930	200
Air aspiration	Out	8	10	200	20
Channel empty	Out	7	10	200	20

^*∗*^Extractor insertion with peristaltic pump.

**Table 2:** Full-factorial design with coded, real values and analytical responses for robustness evaluation of the procedure for flow-batch alkaline hydrolysis.

Experiment	NaOH (mol L^−1^)	Hydrolysis time (min)	Temperature (°C)	Peak area of total SA^a^
1	−1 (0.09)	−1 (4.5)	−1 (70)	5.90
2	1 (0.11)	−1 (4.5)	−1 (70)	5.66
3	−1 (0.09)	1 (5.5)	−1 (70)	7.06
4	1 (0.11)	1 (5.5)	−1 (70)	5.70
5	−1 (0.09)	−1 (4.5)	1 (90)	7.07
6	1 (0.11)	−1 (4.5)	1 (90)	6.31
7	−1 (0.09)	1 (5.5)	1 (90)	5.35
8	1 (0.11)	1 (5.5)	1 (90)	5.90

^a^Mean values of duplicate measurements.

**Table 3:** Contents of free and total SA determined by the proposed and reference procedures (*n* = 3).

Samples	Species	Content (mg kg^−1^)		*F*-values
		Proposed	Reference [16]	
Sugarcane leaves	Free SA	0.29 ± 0.05	0.30 ± 0.02	6.2
	Total SA	0.38 ± 0.01	0.31 ± 0.01	1.0
Corn leaves	Free SA	0.18 ± 0.03	0.16 ± 0.02	2.2
	Total SA	0.89 ± 0.04	0.9 ± 0.1	6.2
Soybean leaves	Free SA	11.4 ± 0.3	11 ± 1	11
	Total SA	12 ± 1	11.2 ± 0.3	11

*F*_critical_ (95% confidence level): 19.

## Data Availability

The data used to support this study are available from the corresponding author upon request.

## Supplementary Materials

Supplementary Materials Supplementary material includes further information on the optimization of the alkaline hydrolysis (full-factorial design, Table S1) and of SA extraction (Doehlert design, Table S2) and ANOVA parameters for the quadratic model (Table S3).
