# Supplementary material for: Flow-Batch Sample Preparation for Fractionation of the Stress Signaling Phytohormone Salicylic Acid in Fresh Leaves
Source: J Anal Methods Chem. 2020 Jul 17;2020:8865849. doi: 10.1155/2020/8865849 (PMC7382727; doi:10.1155/2020/8865849)
Supplement: Supplementary Materials — Supplementary material includes further information on the optimization of the alkaline hydrolysis (full-factorial design, Table S1) and of SA extraction (Doehlert design, Table S2) and ANOVA parameters for the quadratic model (Table S3). [file 8865849.f1.docx]

**Supplementary material**

Table S1: Full factorial design with coded, real values, and analytical responses related to optimization of the flow-batch alkaline hydrolysis.

| Experiment | Hydrolysis time (s) | NaOH  (mol L^-1^)^a^ | Temperature  (°C) | Peak area of total SA^b^ |
| --- | --- | --- | --- | --- |
| 1 | -1 (30) | -1 (0.025) | -1 (40) | 0.25 |
| 2 | 1 (300) | -1 (0.025) | -1 (40) | 0.97 |
| 3 | -1 (30) | 1 (0.100) | -1 (40) | 0.64 |
| 4 | 1 (300) | 1 (0.100) | -1 (40) | 1.62 |
| 5 | -1 (30) | -1 (0.025) | 1 (80) | 0.67 |
| 6 | 1 (300) | -1 (0.025) | 1 (80) | 4.05 |
| 7 | -1 (30) | 1 (0.100) | 1 (80) | 2.79 |
| 8 | 1 (300) | 1 (0.100) | 1 (80) | 5.98 |

^a^Concentrations in the sample zone, *i.e.* after dilution in the sample extract;

^b^Mean values of duplicate measurements

Table S2: Doehlert design with coded, real values, and analytical responses for optimization of SA extraction.

| Experiment | Extraction time  (min) | Microwave power  (%) | Peak Areas^a^ | |
| --- | --- | --- | --- | --- |
|  |  |  | Free SA | Total SA |
| 1 | 1 (8) | 0 (40) | 3.43 | 9.01 |
| 2 | 0.5 (6.5) | 0.866 (60) | 3.28 | 8.50 |
| 3 | -1 (2) | 0 (40) | 3.09 | 6.56 |
| 4 | -0.5 (3.5) | -0.866 (20) | 3.23 | 10.04 |
| 5 | 0.5 (6.5) | -0.866 (20) | 3.47 | 13.24 |
| 6 | -0.5 (3.5) | 0.866 (60) | 3.10 | 8.67 |
| 7^b^ | 0 (5) | 0 (40) | 3.45, 3.12, 3.30 | 9.15, 10.11, 9.70 |

^a^Mean values of duplicate measurements; ^b^Central point replicates

Table S3: ANOVA parameters for the quadratic model fitted from area of total SA at
95 % confidence level.

| Parameter | Sum of Squares (SS) | Degree of freedom (df) | Mean of Square (MS) | *F*_cal_ | *F*_tab_ |
| --- | --- | --- | --- | --- | --- |
| Regression | 25 | 5 | 5 | 53.2 | 9.01 |
| Residual | 0.28 | 3 | 0.094 |  |  |
| Lack of fit | 0.056 | 1 | 0.056 | 0.50 | 18.5 |
| Pure error | 0.226 | 2 | 0.113 |  |  |
| Total | 25.28 | 8 | 3.15 |  |  |
| R^2^ | 0.9888 | R | 0.9944 |  |  |
| R^2^ max. | 0.9910 | R max. | 0.9955 |  |  |
